# Case Report: Anti-neural cell adhesion molecule 1 antibody-positive encephalitis presenting with schizophrenia-like symptoms and an ovarian teratoma

**DOI:** 10.3389/fimmu.2026.1587199

**Published:** 2026-01-30

**Authors:** Nanami Yamanaka, Masaya Honda, Hiroki Shiwaku, Saori Toyoda, Yukiko Motokawa, Makoto Hara, Shusaku Tomita, Fumitaka Shimizu, Michiaki Koga, Masayuki Nakamori

**Affiliations:** 1 Department of Neurology and Clinical Neuroscience, Yamaguchi University Graduate School of Medicine, Ube, Yamaguchi, Japan; 2 Department of Psychiatry and Behavioral Sciences, Institute of Science Tokyo University Graduate School, Bunkyo-ku, Tokyo, Japan; 3 Division of Neurology, Department of Medicine, Nihon University School of Medicine, Itabashi-ku, Tokyo, Japan; 4 National Hospital Organization Kanmon Medical Center, Shimonoseki, Yamaguchi, Japan; 5 Faculty of Medicine and Health Sciences, Yamaguchi University Graduate School of Medicine, Ube, Yamaguchi, Japan

**Keywords:** anti-NCAM1 antibody, autoimmune encephalitis, case report, neural cell adhesion molecule 1, ovarian teratoma

## Abstract

Autoimmune encephalitis (AE) has been linked to various autoantibodies, with anti-neural cell adhesion molecule 1 (NCAM1) antibodies recently identified in patients with schizophrenia. Their pathogenic potential has been demonstrated in passive transfer experiments in mice. We report the first case of AE associated with anti-NCAM1 antibodies in a 15-year-old female who presented with acute-onset psychiatric symptoms, including delusions, hallucinations, and catalepsy, along with fever and impaired consciousness. Cerebrospinal fluid analysis showed a high IgG index, and electroencephalography revealed generalized slow-wave activity. However, brain magnetic resonance imaging was unremarkable. A pelvic tumor was identified, and adnexectomy confirmed a right mature cystic teratoma. Given the clinical presentation, anti-N-methyl-D-aspartate receptor (NMDAR) encephalitis was initially suspected, and the patient was treated with intravenous methylprednisolone and intravenous immunoglobulin, resulting in significant improvement. Despite a subsequent relapse of hallucinations, additional immunotherapy led to complete resolution of symptoms. Notably, anti-NMDAR and other well-known neuronal surface antibodies were negative in both serum and cerebrospinal fluid, whereas anti-NCAM1 antibodies were detected. Immunohistochemical analysis confirmed NCAM1 expression in the excised ovarian tissue, and post-treatment serum tests showed the disappearance of anti-NCAM1 antibodies. The concurrent resolution of clinical symptoms and the disappearance of these antibodies support their pathogenic role. The clinical presentation and paraneoplastic association with teratoma closely resemble anti-NMDAR encephalitis, suggesting that anti-NCAM1 antibodies may define a novel form of AE with psychotic manifestations. Further research is needed to clarify their role in neuroinflammatory disorders.

## Introduction

Recently, several autoantibodies associated with autoimmune encephalitis (AE) have been identified. When targeting synaptic surface antigens, immunotherapy is often effective, highlighting the importance of accurate diagnosis. Anti-N-methyl-D-aspartate receptor (NMDAR) encephalitis is a well-recognized example and is one of the most frequently diagnosed autoimmune encephalitis, particularly in young females. This disorder is characterized by prominent psychiatric symptoms, which often appear early in the disease course and may lead to misdiagnosis as a primary psychiatric disorder. Early recognition and prompt initiation of immunotherapy are crucial for improving outcomes and minimizing long-term cognitive impairment (1).

In 2022, a novel autoantibody against neural cell adhesion molecule 1 (NCAM1) was identified in patients with schizophrenia, and its pathogenicity was confirmed through passive transfer to mice (2). However, as these patients exhibited a chronic course without signs of inflammation, whether anti-NCAM1 autoantibodies contribute to encephalitis remains unclear.

Here, we report the first documented case of autoimmune encephalitis associated with anti-NCAM1 antibodies, characterized by schizophrenia-like symptoms and an ovarian teratoma. This case expands our understanding of autoimmune encephalitis and its treatment.

## Case description

A 15-year-old female with no medical history presented with a 5-day history of delusions, hallucinations, insomnia, stereotypy, and catalepsy, followed by fever (38.4 °C) on the second day of illness. No preceding infection was noted. Electroencephalography (EEG) revealed generalized slow-wave activity at 3–4 Hz without spikes or sharp waves. She was subsequently admitted to our hospital because of suspected limbic encephalitis.

Upon admission, the patient’s body temperature was 37.8 °C. She scored 3A on the Japan Coma Scale and E4V3M1 on the Glasgow Coma Scale. She was unresponsive, with her eyes open, but neither tracked objects nor blinking in response to visual threats. Notably, she engaged in pessimistic, self-reflective self-talk. She exhibited catalepsy, and her muscle strength and tendon reflexes were normal with no Babinski signs. Her arms tremored intermittently at a frequency of 3–4 Hz, although no faciobrachial dystonic seizures were observed.

Blood test results revealed no significant abnormalities. A cerebrospinal fluid (CSF) analysis showed a white blood cell count of 1 cell/μL (100% lymphocytes), protein 15.7 mg/dL, IgG index 0.75 (normal < 0.7), and IL-6 3.8 pg/mL, with negative findings for oligoclonal band. A Film Array^®^ meningitis/encephalitis panel (including *Escherichia coli K1*, *Haemophilus influenzae*, *Listeria monocytogenes*, *Neisseria meningitidis*, *Streptococcus agalactiae*, *Streptococcus pneumoniae*, Cytomegalovirus, Enterovirus, Herpes simplex virus 1, Herpes simplex virus 2, Human herpesvirus 6, Human parechovirus, Varicella zoster virus, and *Cryptococcus neoformans/gattii*) using the CSF was negative. The repeat cytology and culture results were negative. Magnetic resonance imaging (MRI) revealed no abnormalities.

Given the acute-onset psychotic symptoms, altered consciousness, fever, and catalepsy, autoimmune encephalitis with anti-NMDAR antibodies is the primary differential diagnosis (3). Computed tomography (CT) of the trunk revealed an internally heterogeneous tumor measuring 48 mm with fatty components in the pelvic cavity. Pelvic contrast-enhanced MRI suggested that the tumor was an ovarian teratoma. On the day of admission, right adnexectomy was performed, and a pathological examination confirmed a mature cystic teratoma.

## Method

### Cell-based assay and immunohistochemistry

The neuronal surface antibodies, including those targeting NMDAR, AMPA receptor, leucine-rich glioma-inactivated 1 (LGI1), contactin-associated protein 2 (Caspr2), GABA_A_ receptor, and GABA_B_ receptor, were tested using a commercial fixed HEK293 cell–based assay (CBA) (EUROIMMUN BIOCHIP kit). The CBA, along with the tissue-based assay using snap-frozen sections of rat brain and the live neuron assay using cultured E18 rat hippocampal cells, followed the methodology previously described by Hara et al. (4).

Cell-based assay for anti-NCAM1 antibody detection using HeLa cells transiently transfected with a plasmid encoding full-length human NCAM1 and EGFP as a transfection marker. HeLa cells were fixed for 30 min at room temperature in 2% paraformaldehyde (prepared in phosphate buffer), treated with 0.1% Triton X-100 in PBS, and blocked with PBS containing 10% FBS. The cells were then incubated with patient serum or primary antibody diluted in blocking buffer. For the cell-based assay, serum with an autoantibody titer ≥ 1:30 and CSF with an autoantibody titer ≥ 1:2 were defined as positive for autoantibodies. The following antibodies were used for immunocytochemistry and immunohistochemistry: Anti-NCAM1 antibody (1:200, MA5-11563, Thermo Scientific, Waltham, MA, USA), Cy3-conjugated anti-human IgG (1:500, 709-165-149, Jackson Laboratory, Bar Harbor, ME, USA), Alexa Fluor 488-conjugated anti-rabbit IgG (1:500, A21206, Thermo Scientific, Waltham, MA, USA).

Nuclei were stained with DAPI (0.2 μg/ml in PBS; DOJINDO), and images were acquired using an Olympus FV1200 confocal microscope (Tokyo, Japan). The immunostaining techniques for ovarian teratoma followed the methodology described by Shiwaku et al. (2).

### Treatment course

**Figure 1:**
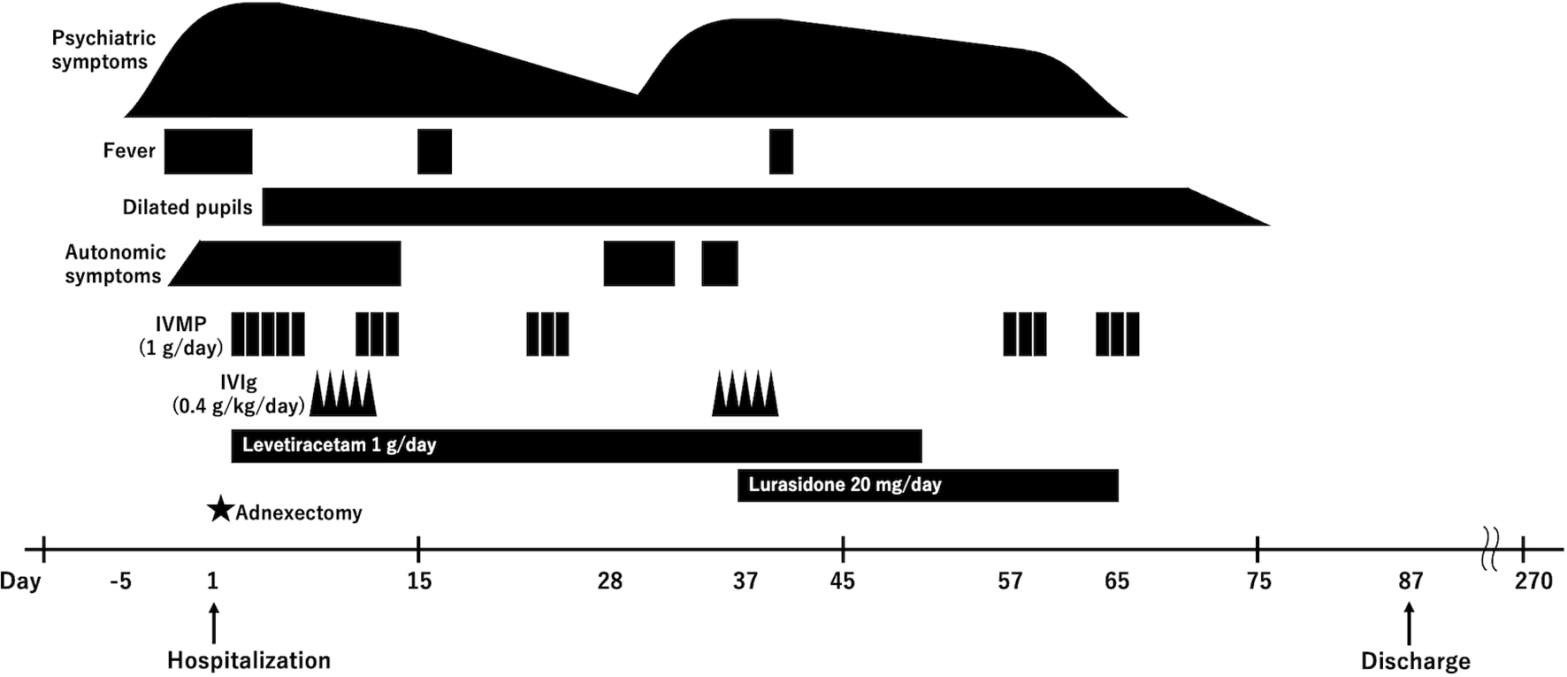
Clinical course. Following hospitalization, the patient underwent right adnexectomy due to suspected anti-NMDAR encephalitis, which revealed a mature cystic teratoma. The patient received three courses of IVMP and one course of IVIg, which led to an improvement in both consciousness and psychotic symptoms. However, hallucinations and autonomic symptoms recurred during the treatment. Although a second course of IVIg was administered with a limited effect, two additional courses of IVMP successfully resolved the psychotic symptoms, allowing the patient to be discharged. The patient remained in remission for more than five months without requiring further treatment. IVMP, intravenous methylprednisolone; IVIg, intravenous immunoglobulins.

The patient was started on intravenous methylprednisolone (IVMP) at 1 g/day from the day after admission (day 1). As transient fluctuations in consciousness at admission were clinically interpreted as acute symptomatic seizures, levetiracetam (1 g/day) was initiated as prophylactic therapy. After several days of IVMP treatment, involuntary movements in the right upper limb and the catalepsy improved. The patient began making eye contact and showed an increase in purposeful speech. Within a few more days, she responded to commands, although self-talk persisted. Additional symptoms, such as excessive salivation, sweating, and dilated pupils (6/6 mm, light reflex: prompt), were also noted (**Figure 1**).

Following administration of intravenous immunoglobulin (IVIg) at 0.4 g/kg/day and completion of the second and third courses of IVMP, her condition showed further improvement. EEG findings were nearly normalized, and her cognitive test scores, including the Mini-Mental State Examination, Frontal Assessment Battery, and Trail Making Test, gradually improved.

**Figure 2:**
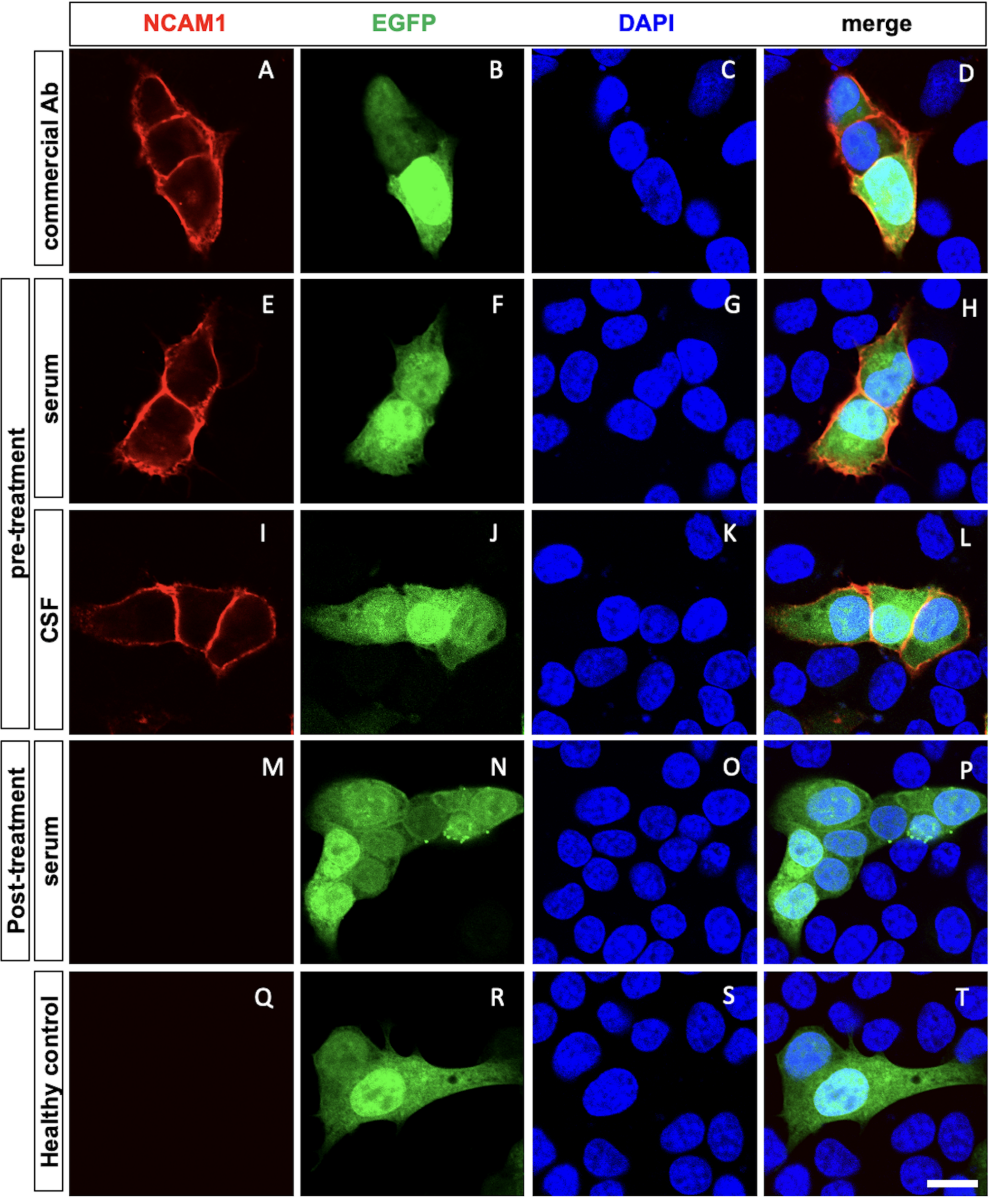
A cell-based assay shows anti-NCAM1 antibody in pre-treatment serum and cerebrospinal fluid. Confocal images of cell-based assays using HeLa cells expressing NCAM1 (red), EGFP (green, transfected-cell marker), and DAPI (blue, nuclei). NCAM1 and EGFP were expressed in the plasmids under each condition. Positive control staining using a commercial anti-NCAM1 antibody **(A–D)**. Anti-NCAM1 antibodies present in the pre-treatment serum and CSF bound to the membrane of EGFP-positive cells **(E-L)**, while post-treatment serum showed no binding **(M-P)**. No anti-NCAM1 antibodies were detected in the age- and sex-matched healthy controls **(Q-T)**. Scale bar: 10 µm. Ab, antibody; CSF, Cerebrospinal fluid; NCAM1, neural cell adhesion molecule 1; EGFP, enhanced green fluorescent protein; DAPI, 4’,6-diamidino-2-phenylindole.

On day 28, the patient experienced relapse of psychotic symptoms, including insomnia, auditory and tactile hallucinations, delusional experiences, and catalepsy. MRI, EEG, and CSF analyses performed at the time showed no abnormalities. The neuronal surface antibody panel, including antibodies against NMDAR, AMPA receptor, LGI1, Caspr2, GABA_A_ receptor, and GABA_B_ receptor, was negative in both the patient’s serum and CSF in commercial fixed HEK293 CBA (EUROIMMUN BIOCHIP). In addition, the tissue-based assay using frozen sections of rat brain did not show a characteristic neuropil staining pattern, and the live neuron assay using cultured E18 rat hippocampal cells did not show any specific reactivity (**Supplementary Figure**). However, anti-neural cell adhesion molecule 1 (NCAM1) antibodies were detected in both the patient’s serum and CSF (**Figures 2A-L**). The anti-NCAM1 antibody titers, measured by ELISA at the Institute of Science Tokyo, were undetectable 0.12 (A_405_) in CSF, compared with 0.10 (A_405_) in a healthy control. In addition, serum testing for anti-GRP78 antibodies was also negative.

Initiation of a second course of IVIg and administration of lurasidone hydrochloride (20 mg/day) did not result in significant improvement. However, a fourth course of IVMP led to a rapid reduction in the hallucinations. After the fifth course of IVMP, the psychotic symptoms completely resolved, and the patient returned to her pre-illness baseline. No recurrence of symptoms was observed thereafter, and the patient was discharged home on day 87. Post-treatment serum testing confirmed the disappearance of anti-NCAM1 antibodies (**Figures 1A, M-P**).

### The histopathological analysis of ovarian teratoma

Histopathological examination revealed a mature cystic teratoma composed of well-differentiated glial tissue, ganglion cells, peripheral nerves, adipose tissue, smooth muscle, sweat glands, and hair follicles. No immature, malignant, or inflammatory components were identified in the stroma.

NCAM1-positive cells, which elicited an autoantibody response in the pre-treatment serum. In contrast, no such response was observed with the post-treatment serum or the serum from age- and sex-matched healthy controls (**Figure 1B**).

Footnote: The report adheres to the CARE checklist for case reports. Informed consent for publication was provided by both the patient and their legal guardian.

## Discussion

This is the first clinical report describing autoimmune encephalitis in which anti-NCAM1 antibodies may have been involved. NCAM1 is a multidomain protein with several immunoglobulin-like domains and fibronectin III domains (5) and plays a significant role in neuronal migration, axonal growth, synaptogenesis, and synaptic plasticity, all of which are essential for the proper nervous system function (6, 7). Autoantibodies against NCAM1 and neurexin 1α (NRXN1) have been identified in the serum and/or CSF of patients with schizophrenia. In animal models, the administration of purified patient IgG to mice induces cognitive impairments, suggesting a potential role in neurological pathogenesis (2, 8). Specifically, intrathecal administration of anti-NCAM1 antibodies leads to decreased synaptic density and schizophrenia-related behaviors in mice (2). These findings suggest that anti-NCAM1 antibodies can affect the synaptic function. Anti-NCAM1 antibodies target the extracellular immunoglobulin 1 domain of NCAM1, which is located on the cell surface and expressed on neurons and glia (2). Based on these observations, it has been hypothesized that anti-NCAM1 antibodies could theoretically contribute to autoimmune encephalitis. However, no such cases have been reported until now.

In the present case, the patient’s symptoms were primarily psychotic, with schizophrenia-like hallucinations and delusions, consistent with the role of anti-NCAM1 antibodies. Furthermore, the disappearance of serum anti-NCAM1 antibodies following immunotherapy suggests a temporal association between antibody levels and disease activity (**Figures 2E-P**). Although these observations do not establish causality, they support the possibility that anti-NCAM1 antibodies were involved in the disease process.

From the standpoint of balancing sensitivity and specificity, the human antigen–based CBA remains the most reliable method for detecting neuronal surface antibodies (9). Despite the detection of anti-NCAM1 antibodies using a fixed CBA, tissue-based assays (TBA) employing rat brain sections did not reveal a characteristic neuropil staining pattern. Although negative TBA or live-neuron assay results have been described in other neuronal antibody studies, such as those involving anti-GlyR antibodies, the underlying reasons vary depending on the tissue localization. In addition, anti-MOG antibodies recognize glial cell surface antigens, but their detection sensitivity can be reduced by species differences between humans and rodents (10, 11). Reliable comparative data indicate that the Ig1 domain of NCAM1, which serves as the primary epitope for anti-NCAM1 antibodies, differs by five amino acids between humans and rodents out of 92 residues. In addition, the relatively low NCAM1 antibody titer in the CSF may have contributed to the absence of reactivity in TBA and live-neuron assays, despite positive results in CBA. Therefore, the lack of specific staining in tissue- or live-neuron assays under the current conditions does not necessarily exclude the involvement of anti-NCAM1 antibodies.

The clinical presentation, including the rapid progression of psychotic symptoms over three months, abnormal EEG findings (generalized 3-to 4-Hz slow waves), elevated IgG index, presence of a mature ovarian teratoma, and a favorable response to steroid treatment, closely mirrors that of NMDAR encephalitis. This case met the criteria for probable NMDAR encephalitis based on the diagnostic guidelines of Graus et al. (3), although anti-NMDAR antibodies were negative.

Anti-NMDAR encephalitis is a prototypical form of AE characterized by psychiatric symptoms, with a well-established pathogenicity of anti-NMDAR antibodies and their association with teratomas. The first case of anti-NMDAR encephalitis associated with treatment-responsive paraneoplastic encephalitis and ovarian teratoma was reported in 2007 (12). Approximately 90% of anti-NMDAR encephalitis cases present with psychiatric or behavioral symptoms in the early phase (1), as anti-NMDAR antibodies downregulate receptors and impair NMDAR-mediated currents (13, 14). Ovarian teratomas in anti-NMDAR encephalitis contain NMDAR-expressing neurons and B-cell infiltration (5, 15–17). In contrast, the relationship between NCAM1 expression and ovarian teratomas has not been previously explored.

**Figure 3:**
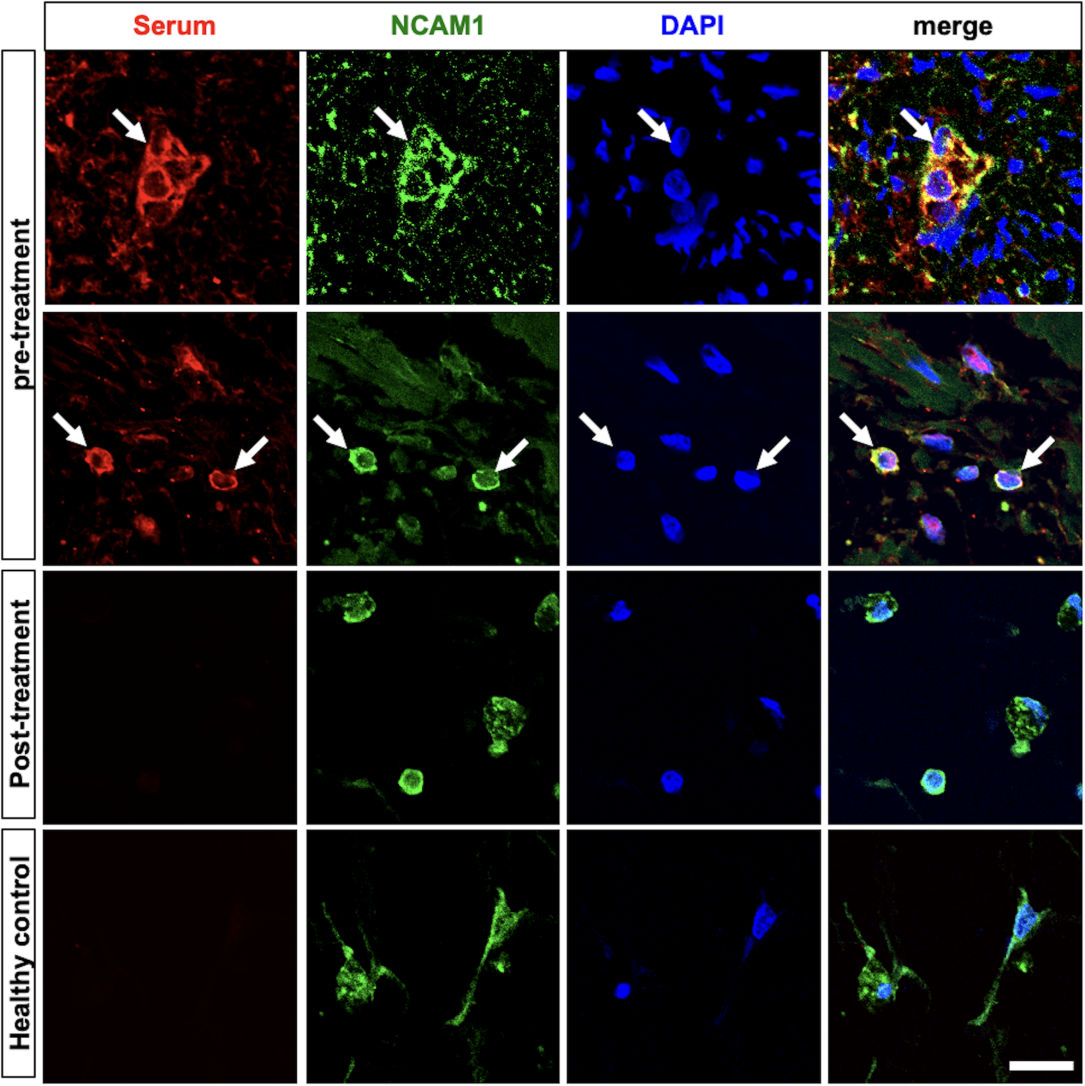
Immunofluorescent staining of ovarian teratoma tissue. Pre-treatment serum from the patient (red) and staining with a commercial anti-NCAM1 antibody (green) were used, with nuclear staining with DAPI (blue). The merged image shows an overlap between the patient’s serum and the commercial anti-NCAM1 antibody, confirming the presence of NCAM1 on the surface of the teratoma cells, indicating that the patient’s serum antibodies target NCAM1 (arrows). No binding to NCAM1 was observed in post-treatment serum or age- and sex-matched healthy control serum. Scale bar: 10 µm. NCAM1, neural cell adhesion molecule 1; DAPI, 4’,6-diamidino-2-phenylindole.

In the present case, a histopathological examination of the excised ovarian teratoma revealed NCAM1-positive cells along with an autoantibody response directed against them (**Figure 3**). This finding raises the possibility that a paraneoplastic mechanism, analogous to that proposed for anti-NMDAR encephalitis, may have contributed to disease development. In line with this hypothesis, tumor removal may have played a role in clinical improvement. In addition, although serum anti-GRP78 antibodies were negative, other tumor-related factors may have contributed to blood–brain barrier dysfunction, facilitating antibody access to the central nervous system. Similar paraneoplastic mechanisms have been described in other autoimmune neurological disorders (18, 19).

Several limitations should be acknowledged. First, this report describes a single clinical case, and the pathogenic role of anti-NCAM1 antibodies in humans has not yet been established, precluding definitive conclusions regarding disease mechanisms. Second, although anti-NCAM1 antibodies were detected by ELISA and fixed cell-based assay (CBA), their presence could not be confirmed by independent assays such as tissue-based immunohistochemistry or live-neuron assays, as standardized and validated conditions for detecting anti-NCAM1 antibodies are not yet available. Third, interpretation of ELISA-measured antibody titers remains challenging because cutoff values and the clinical significance of absolute levels are undefined. In addition, the epitope structure and IgG subclass composition were not fully characterized, and live CBA was not performed.

Despite these limitations, this case highlights a previously unreported clinical scenario in which anti-NCAM1 antibodies may be associated with autoimmune encephalitis mimicking anti-NMDAR encephalitis. This report expands the potential clinical spectrum related to anti-NCAM1 antibodies and underscores the need for further case accumulation and methodological refinement.

## Data Availability

The original contributions presented in the study are included in the article/**Supplementary Material**. Further inquiries can be directed to the corresponding author.
