# Supplementary material for: Case Report: Anti-neural cell adhesion molecule 1 antibody-positive encephalitis presenting with schizophrenia-like symptoms and an ovarian teratoma
Source: Front Immunol. 2026 Jan 30;17:1587199. doi: 10.3389/fimmu.2026.1587199 (PMC12901500; doi:10.3389/fimmu.2026.1587199)
Supplement: Supplementary file 1 [file DataSheet1.pdf]

CARE case report guidelines

CARE Checklist of information to include when writing a case report

(cc) BY-NC-ND

Topic	Item	Checklist item description	Reported on Line
**Title**	1	The diagnosis or intervention of primary focus followed by the words "case report"	Title
**Key Words**	2	2 to 5 key words that identify diagnoses or interventions in this case report, including "case report"	Keywords
**Abstract (no references)**	3a	Introduction: What is unique about this case and what does it add to the scientific literature?	Abstract
	3b	Main symptoms and/or important clinical findings	Abstract
	3c	The main diagnoses, therapeutic interventions, and outcomes	Abstract
	3d	Conclusion—What is the main "take-away" lesson(s) from this case?	Abstract
**Introduction**	4	One or two paragraphs summarizing why this case is unique (**may include references**)	Introduction
**Patient Information**	5a	De-identified patient specific information.	Case description
	5b	Primary concerns and symptoms of the patient.	Case description
	5c	Medical, family, and psycho-social history including relevant genetic information	Case description
	5d	Relevant past interventions with outcomes	Case description
**Clinical Findings**	6	Describe significant physical examination (PE) and important clinical findings.	Case description
**Timeline**	7	Historical and current information from this episode of care organized as a timeline	Figure 1
**Diagnostic Assessment**	8a	Diagnostic testing (such as PE, laboratory testing, imaging, surveys).	Case description and Method
	8b	Diagnostic challenges (such as access to testing, financial, or cultural)	Discussion
	8c	Diagnosis (including other diagnoses considered)	Case description
	8d	Prognosis (such as staging in oncology) where applicable	Treatment course
**Therapeutic Intervention**	9a	Types of therapeutic intervention (such as pharmacologic, surgical, preventive, self-care)	Treatment course
	9b	Administration of therapeutic intervention (such as dosage, strength, duration)	Treatment course
	9c	Changes in therapeutic intervention (with rationale)	Treatment course
**Follow-up and Outcomes**	10a	Clinician and patient-assessed outcomes (if available)	Treatment course
	10b	Important follow-up diagnostic and other test results	Treatment course
	10c	Intervention adherence and tolerability (How was this assessed?)	Not applicable
	10d	Adverse and unanticipated events	Not reported
**Discussion**	11a	A scientific discussion of the strengths AND limitations associated with this case report	Discussion
	11b	Discussion of the relevant medical literature **with references**.	Discussion
	11c	The scientific rationale for any conclusions (including assessment of possible causes)	Discussion
	11d	The primary "take-away" lessons of this case report (without references) in a one paragraph conclusion	Discussion
**Patient Perspective**	12	The patient should share their perspective in one to two paragraphs on the treatment(s) they received	Not applicable
**Informed Consent**	13	Did the patient give informed consent? Please provide if requested	**Yes** ☑ **No** ☐
