# Supplementary material for: Case Report: Anti-neural cell adhesion molecule 1 antibody-positive encephalitis presenting with schizophrenia-like symptoms and an ovarian teratoma
Source: Front Immunol. 2026 Jan 30;17:1587199. doi: 10.3389/fimmu.2026.1587199 (PMC12901500; doi:10.3389/fimmu.2026.1587199)
Supplement: Supplementary file 2 [file Table1.docx]

**Supplementary material**

- **Supplementary Figure 1.**

**Screening results of the patient’s cerebrospinal fluid using tissue-based and live-neuron assays.**


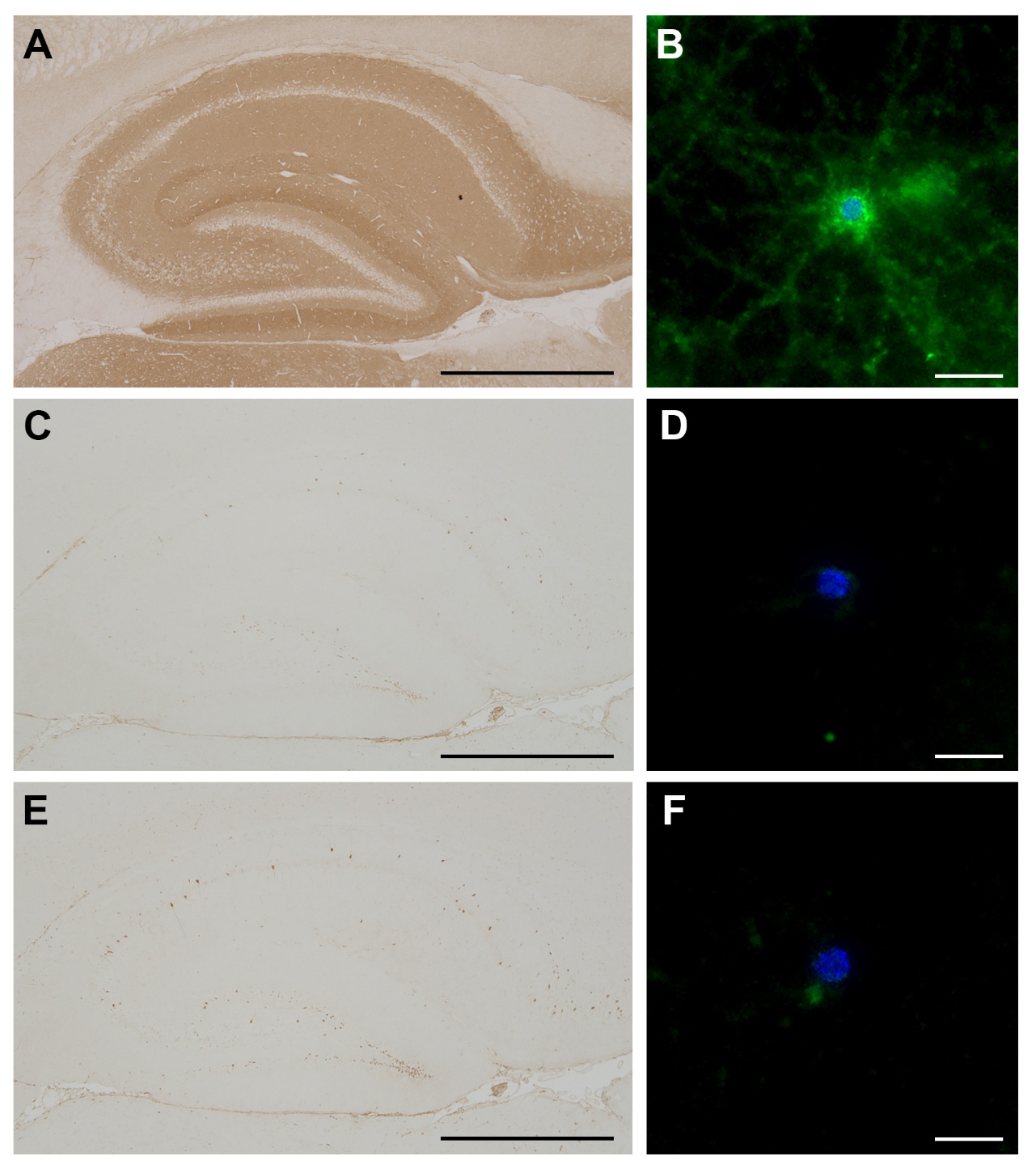


The positive control cerebrospinal fluid (CSF) containing anti–leucine-rich glioma-inactivated 1 antibodies showed distinct immunolabeling in the hippocampal neuropil on the tissue-based assay (A) and robust immunofluorescent labeling on synapses of cultured hippocampal live neurons (B, green). In contrast, the negative control CSF exhibited no detectable immunolabeling in hippocampal tissue (C) or on synapses of live neurons (D). The CSF from the present patient likewise demonstrated no immunolabeling on either hippocampal tissue (E) or cultured hippocampal live neurons (F). In the live-neuron assay, nuclei were counterstained with DAPI (blue in B, D, and F). Scale bars: 1 mm (A, C, E) and 20 µm (B, D, F).

- **Supplementary methods**

**1. In-house tissue-based assays for screening neuronal surface antibodies**

The tissue-based assay, consisting of immunohistochemistry on rat brain tissue, was performed as previously described (1, 2). Briefly, adult female Wistar rats were sacrificed without perfusion, and the brains were removed and fixed in 4% paraformaldehyde for 1 hour at 4 °C. Tissue was cryoprotected in 40% sucrose for 48 hours, embedded in a freezing compound, and snap-frozen in isopentane chilled with liquid nitrogen. Six-micrometer-thick coronal sections were sequentially incubated with 0.3% hydrogen peroxide for 15 minutes, followed by 5% goat serum for 1 hour, and then with patient or control cerebrospinal fluid (CSF; 1:2) or serum (1:200) overnight at 4 °C. After incubation with biotinylated secondary antibodies against human IgG (1:2000; BA-3000, Vector), immunoreactivity was visualized using the avidin–biotin–peroxidase method.

**2. In-house live-neuron assay for screening neuronal surface antibodies**

Primary rat hippocampal neuronal cultures were prepared as previously reported (1, 2). Mature live neurons grown on coverslips were incubated with patient or control CSF (1:2) for 1 hour at 4 °C. After removal of the incubation medium and extensive washing with phosphate-buffered saline (PBS), neurons were fixed with 4% paraformaldehyde and immunolabeled with Alexa Fluor^®^ 488–conjugated goat anti-human IgG (1:1000; A11013, Invitrogen). Images were acquired using a fluorescence microscope (BZ-X810, KEYENCE, Osaka, Japan).

**3. Cell-based assay using a commercially available panel**

Given the small possibility of false-negative findings with these screening modalities, we additionally performed a commercially available cell-based assay targeting nine neuronal surface antigens. These included the *N*-methyl-D-aspartate receptor, α-amino-3-hydroxy-5-methyl-4-isoxazolepropionic acid receptor, leucine-rich glioma-inactivated 1, contactin-associated protein-like 2, γ-aminobutyric acid type B receptor, dipeptidyl-peptidase-like protein 6, γ-aminobutyric acid type A receptor, IgLON family member 5, and metabotropic glutamate receptor 5. The assay was conducted using the BIOCHIP platform (EUROIMMUN, Lübeck, Germany) to confirm the absence of neuronal surface antibodies.

- **Supplementary references**

1. Mizoguchi T, Hara M, Hirose S, et al. Novel qEEG Biomarker to Distinguish Anti-NMDAR Encephalitis From Other Types of Autoimmune Encephalitis. Front Immunol 2022;13:845272.
2. Kataoka H, Hara M, Nanaura H, et al. Antibodies against unknown neuronal antigens following herpes simplex virus encephalitis: A report of three patients. Brain Disorders 2025;19:100260.
